# Inhibitory effects of water chestnut (*Trapa bispinosa* Roxb.) extract plus lutein on the progression of cataract and their impact on the retina and aorta in diabetic rats

**DOI:** 10.1007/s40200-025-01845-9

**Published:** 2026-01-14

**Authors:** Hiroshi Mizuno, Gaku Ishigooka, Denan Jin, Shinji Takai, Teruyo Kida

**Affiliations:** 1 Department of Ophthalmology, Osaka Medical and Pharmaceutical University, 2-7 Daigaku-machi, Takatsuki, Osaka, Japan; 2 Department of Innovative Medicine, Osaka Medical and Pharmaceutical University, Osaka, Japan

**Keywords:** *Trapa bispinosa* Roxb. extract, Lutein, Diabetic cataract, Oxidative stress, Antioxidant

## Abstract

**Purpose:**

*Trapa bispinosa* Roxb. extract (TBE), combined with lutein, has an inhibitory effect on cataract progression. However, no prior studies evaluated oxidative stress- and antioxidant-related factors simultaneously. This study aimed to investigate TBE plus lutein’s potential mechanism to suppress cataract progression and examine its effects on retinal tissue and aorta in spontaneous diabetic Torii (SDT) rats.

**Methods:**

Seven-week-old SDT rats (*n* = 10) and Sprague-Dawley (SD) control rats (*n* = 5) were divided into three groups: SDT-administered TBE plus lutein (TBE plus lutein), untreated SDT, and control. Blood glucose levels and anterior segment photographs were obtained. Subsequently, polymerase chain reaction (PCR) was used to measure the expression of oxidative stress- and antioxidant-related genes in the lenses, retinas, and aortas. Immunohistochemical staining of the lenses was performed to detect the expression of NOX-1 (an oxidative stress-related factor), 4-HNE (a lipid peroxidation product), and CEL (an advanced glycation end product [AGE]).

**Results:**

The progression of diabetic cataract was suppressed in the TBE plus lutein group. PCR results revealed that NOX-1 in lenses was significantly suppressed in the TBE plus lutein group (*p* < 0.05), whereas antioxidant-related factors, such as SOD, GPX, and Peroxiredoxin 6, were significantly increased (*p* < 0.01). A similar trend in NOX-1 expression was observed in the retinas and aortas, but the difference was not significant. Immunohistochemical staining of lenses revealed reduced NOX-1, 4-HNE, and CEL expression in the lenses of the TBE plus lutein group.

**Conclusion:**

TBE plus lutein may inhibit the progression of diabetic cataract by increasing antioxidant-related factors rather than suppressing oxidative stress-related factors.

## Introduction

Cataract is a leading cause of vision loss and blindness, primarily due to aging. However, it can also result from congenital conditions, eye trauma, inflammation, and diseases such as glaucoma and diabetes. Additionally, cataract development is associated with steroid use and certain types of radiation exposure [1]. Hyperglycemia is the primary cause of cataracts and other vascular complications in individuals with diabetes, often leading to retinopathy and eventual vision loss [2]. Both oxidation and glycation are believed to play a role in these processes [3–5]. Glycation refers to the process by which sugars bind to proteins, leading to protein denaturation and the formation of advanced glycation end products (AGEs). AGEs accumulate in the lenses [6] and retina in patients with diabetes [7]. Surgical treatment is the standard for diabetic cataract with vision loss [8]. Eye drops are sometimes prescribed to inhibit the progression of cataracts; however, more effective and simpler treatment options are still needed.

*Trapa bispinosa* Roxb. extract (TBE), the water chestnut, is an annual aquatic grass of the Citrus family that is widely used as a healthy tea in Asia and globally as an edible medicinal plant [9]. Plants of the genus *Trapa* have been reported to have various physiological functions, including antioxidant, antimicrobial, and gastroprotective activities. TBE inhibits the formation of carboxymethyllysine (CML), a key AGE, and suppresses AGE cross-linking in vitro [3].

Lutein is a carotenoid present in the crystalline lenses and macular region of the eye. Notably, it cannot be synthesized in vivo [10]. Lutein intake protects against oxidative stress in the eye, and its dietary intake has been associated with a reduced risk of cataract development. Moreover, it reduces the risk of age-related macular degeneration [11–14].

TBE plus lutein is known to have an inhibitory effect on cataract progression according to previous studies. Many of these studies have investigated the inhibitory effects of TBE and lutein on cataracts using polymerase chain reaction (PCR) to measure antioxidant- and oxidative stress-related factors, alongside histological examinations. However, to the best of our knowledge, no studies have simultaneously evaluated oxidative stress- and antioxidant-related factors in this context. Furthermore, few studies have explored the effects of TBE and lutein on retinal and abdominal aortic complications associated with diabetes. Therefore, in this study, we aimed to investigate the effects of TBE plus lutein on preventing diabetic cataract progression and to clarify the potential underlying mechanism. Additionally, we examined the potential impact of this combination on retinal and abdominal aortic complications associated with diabetes.

## Materials and methods

### Animals

Ten 7-week-old male Spontaneously Diabetic Torii (SDT/Jcl) rats [15, 16] were purchased from CLEA Japan, Inc. (Tokyo, Japan), and five age-matched male SD/Slc (Sprague-Dawley) rats were acquired from Japan SLC, Inc. (Shizuoka, Japan). All rats were housed in an air-conditioned room at approximately 23 °C with 60% humidity. They were maintained on a 12-h light/dark cycle and provided food and water *ad libitum*. The animals were randomly assigned to three experimental groups (*n* = 5 per group): SDT rats administered MF (25 g/day) along with TBE (16 mg/day) and lutein (1.6 mg/day) (TBE + lutein group), SDT rats administered MF alone (25 g/day) (placebo group), and SD rats administered MF (25 g/day; control group). Body weight and blood glucose levels were measured every two weeks. Blood glucose levels were measured using a blood glucose meter purchased from SKK, Co., Ltd. (Aichi, Japan) and assessed by collecting a blood sample from the tip of the rat’s tail. Additionally, anterior segment photographs were obtained every two weeks using slit-lamp examination. All rats were euthanized at 25 weeks of age, and their lenses, retinas, and abdominal aortas were extracted. Animals were handled in accordance with the ARVO Statement for the Use of Animals in Ophthalmic and Vision Research. Our experimental protocols conformed to the Animal Research: Reporting In Vivo Experiments (ARRIVE) guidelines and were approved by the Osaka Medical and Pharmaceutical University Committee on the Use and Care of Animals (approval number: 2019 − 119).

### Chemicals

TBE and lutein were purchased from Santen Pharmaceutical Co. Ltd. (Osaka, Japan). All other chemicals were purchased from Sigma-Aldrich (St. Louis, MO, USA), unless otherwise specified.

### Anesthesia and euthanasia

Each rat was identified by an auricular incision performed under 4% isoflurane anesthesia. Euthanasia was performed by exposure to CO_2_ at the age of 25 weeks [17].

### Evaluation of cataract progression

After euthanasia, the lenses were bluntly extracted using a razor blade via a split corneal incision. The severity of lens opacity was evaluated using a grading scale of 1–4 based on the degree of the cataract [17]. Cataract grading was evaluated visually by placing a grid beneath each extracted lens. Grades were defined as follows: grade 1, the grid was completely visible through the lens; grade 2, the grid was almost completely visible; grade 3, the grid appeared slightly blurred; and grade 4, the grid was completely obscured.

### Real-time reverse transcription polymerase chain reaction (RT-PCR)

Total ribonucleic acid (RNA) was extracted from tissues using TRIzol reagent (Life Technologies, Rockville, MD, USA), dissolved in RNase-free water (Takara Bio Inc., Otsu, Japan), and transcribed into complementary deoxyribonucleic acid using Superscript VIRO software (Invitrogen, Carlsbad, CA, USA). The messenger RNA (mRNA) levels were measured via real-time RT-PCR using Stratagene Mx3000P (Agilent Technologies, San Francisco, CA, USA). Primers for real-time RT-PCR of oxidative stress-related enzymes (NADPH oxidase [NOX]−1, NOX-4, and vascular endothelial growth factor [VEGF]) and antioxidant-related factors (catalase, superoxide dismutase [SOD], glutathione peroxidase [GPX], glyceraldehyde 3-phosphate dehydrogenase [GAPDH], and peroxiredoxin 6) were designed by Roche Diagnostics (Tokyo, Japan). The primers were as follows: 5′-ggcatccctttactgacct-3′ (forward) and 5′-tgctgctcgaatatgaatgg-3′ (reverse) for NOX-1; 5′-gaacccaagttccaagctca-3′ (forward) and 5′-gcacaaaggtccagaaatcc-3′ (reverse) for NOX-4; 5‘-aaaaacgaaagcgcaagaaa-3‘ (forward) and 5’-tttctccgctctgaacaagg-3’ (reverse) for VEGF; 5‘-gcttttgacccaagcaacat-3‘ (forward) and 5’-ggtgagtgtctgggtaagcaa-3’ (reverse) for catalase; 5′-agaaacatggcggtccag-3′ (forward) and 5′-acggacacattggccacac-3′ (reverse) for SOD; 5′-cctaaggcattcctggtatctg-3′ (forward) and 5′-caccrtcatggaaaaacctc-3′ (reverse) for GPX; and 5′-aatgtatccgttgtggatctga-3′ (forward) and 5′-gcttcaccaccttcttgatgt-3′ (reverse) for GAPDH. mRNA levels were normalized to those of GAPDH [17].

### Histological examination

The left eyeballs of all rats were collected after CO_2_ euthanasia, fixed overnight in methanol-based Carnoy’s fixative, and embedded in paraffin. These paraffin blocks were cut into 4-µm-thick cross sections using a microtome (LITORATOMU, REM-710, Yamato Koki Kogyo Ltd., Saitama, Japan). Hematoxylin and eosin (HE) staining was performed on the first section of each group. Subsequent sections were used for immunohistochemical analysis to localize NOX-1 (1:50 dilution, ab131088, Abcam, Cambridge, UK), 4-hydroxy-2-nonenal (4-HNE) (anti-4HNE monoclonal antibody, 1:50 dilution, MHN, JalCa, Shizuoka, Japan), and carboxyethyl lysine (CEL) (1:50 dilution, AGE-M02, Cosmo Bio Co., Ltd., Tokyo, Japan) [17]. Deparaffinized sections were incubated with the respective antibodies overnight at 4 °C, followed by a reaction with components from a labeled streptavidin–biotin peroxidase kit (Dako LSAB kit; Dako, Carpinteria, CA, USA), and the sections were incubated with 3-amino-9-ethylcarbazole for color development, counterstained with hematoxylin, and mounted on cover glasses. All stained slides were examined using an upright light microscope (ECLIPSE Ni; Nikon, Japan) [17].

### Statistical analyses

Normality was assessed using the Shapiro-Wilk test. Data are presented as the mean ± standard error of the mean (SEM). Graphs display SEM unless otherwise indicated. Significant differences among the means of the three groups were evaluated using one-way analysis of variance, followed by Fisher’s exact test. Statistical significance was set at *p* < 0.05. In all graphs, data points without a *p* < 0.05 notation indicate no significant difference. Graphs were created using Microsoft^®^ Excel for Mac ver. 16.78 (23100802).

## Results

### Changes in body weight and blood glucose level

**Fig. 1:**
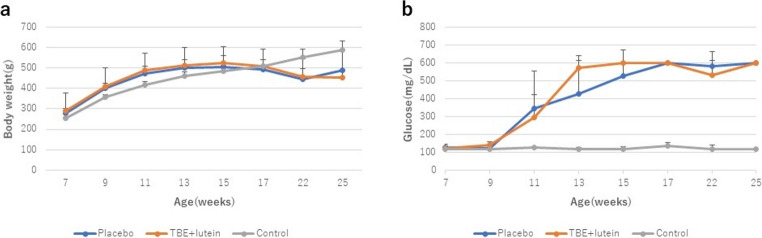
Comparison of body weight and blood glucose levels in SDT and SD rats over time the body weights of SDT rats (TBE + lutein and placebo groups) stabilized at approximately 500 g after 11 weeks of age. In contrast, the body weights of SD rats (control group) continued to increase, reaching approximately 590 g at the time of euthanasia (**a**). The blood glucose levels of SDT rats continued to increase, reaching over 600 mg/dL after 15 weeks of age, while SD rats remained at approximately 120 mg/dL (**b**)

Body weights and blood glucose levels are presented in Fig. 1. The body weights of SDT rats (TBE + lutein and placebo groups) stabilized at approximately 500 g at the age of 11 weeks. In contrast, the body weight of the SD rats (control group) continued to increase, reaching approximately 590 g at the time of euthanasia (Fig. 1a). Blood glucose levels in SDT rats rose steadily, exceeding 600 mg/dL after the age of 15 weeks, whereas levels in SD rats remained at approximately 120 mg/dL (Fig. 1b).

### Cataract progression and its suppression by TBE + lutein

**Fig. 2:**
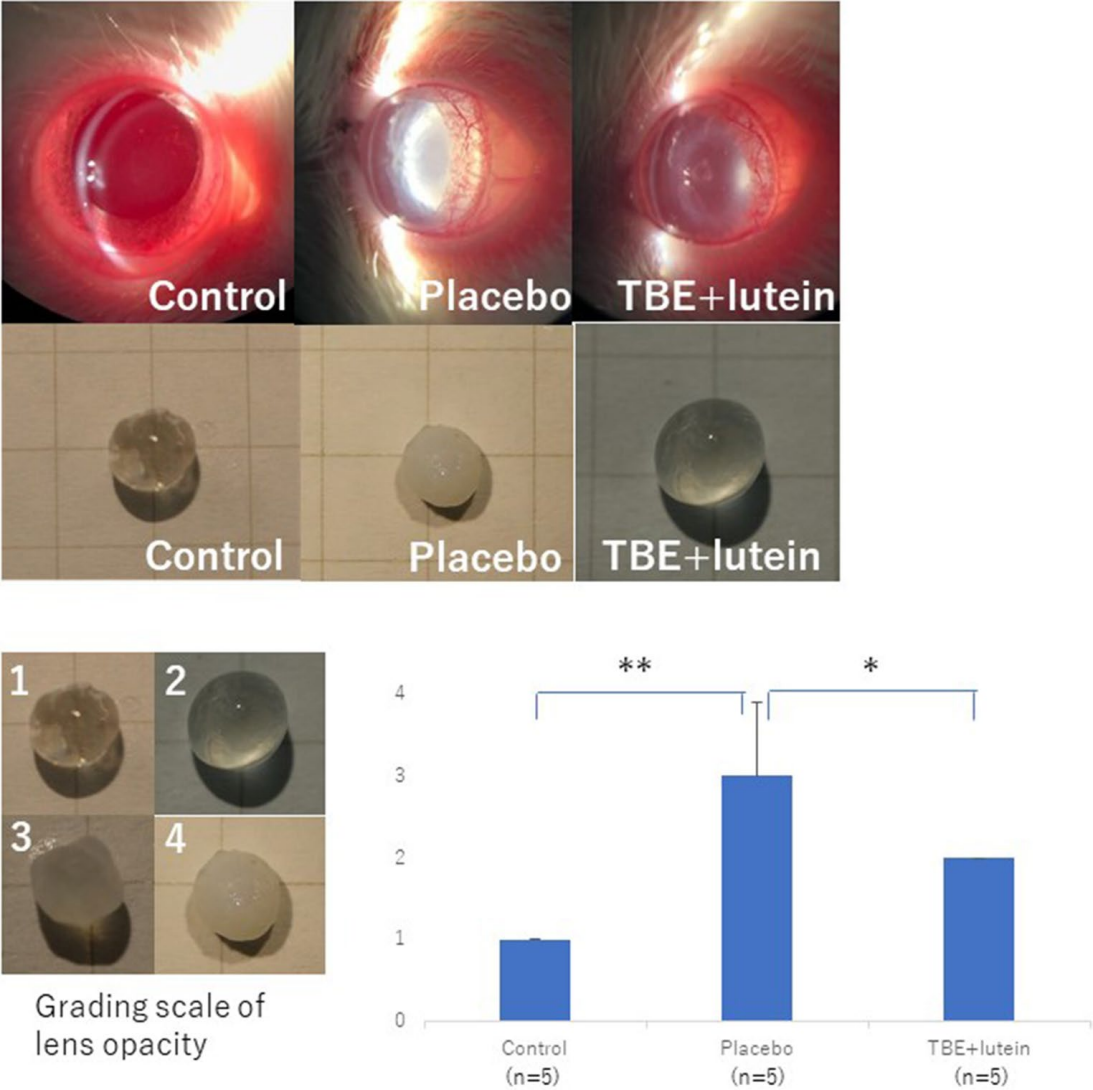
Effect of TBE + lutein supplementation on lens opacity in SDT rats at 25 weeks of age At 25 weeks of age, the lenses of all rats were extracted and rated on a scale of 1–4. The severity of lens opacity in SD rats was consistently grade (1) In contrast, the average severity in the placebo group was grade 3, while the TBE + lutein group exhibited an average opacity of grade (2) Cataract formation significantly progressed in the placebo group compared to that in the control group (*p* < 0.01) and was suppressed in the TBE + lutein group compared to that in the placebo group (*p* < 0.05)

Figure 2 presents lens photographs and severity scores. The severity of lens opacity in SD rats was consistently grade (1) In contrast, the average severity in the placebo group was grade 3, while the TBE + lutein group exhibited an average opacity of grade (2) Cataract formation progressed significantly to a greater extent in the placebo group than in the control group (*p* < 0.01). In contrast, cataract progression was significantly suppressed in the TBE + lutein group than in the placebo group (*p* < 0.05) (Fig. 2).

### RT-PCR results of oxidative stress-related and antioxidant-related factors

**Fig. 3:**
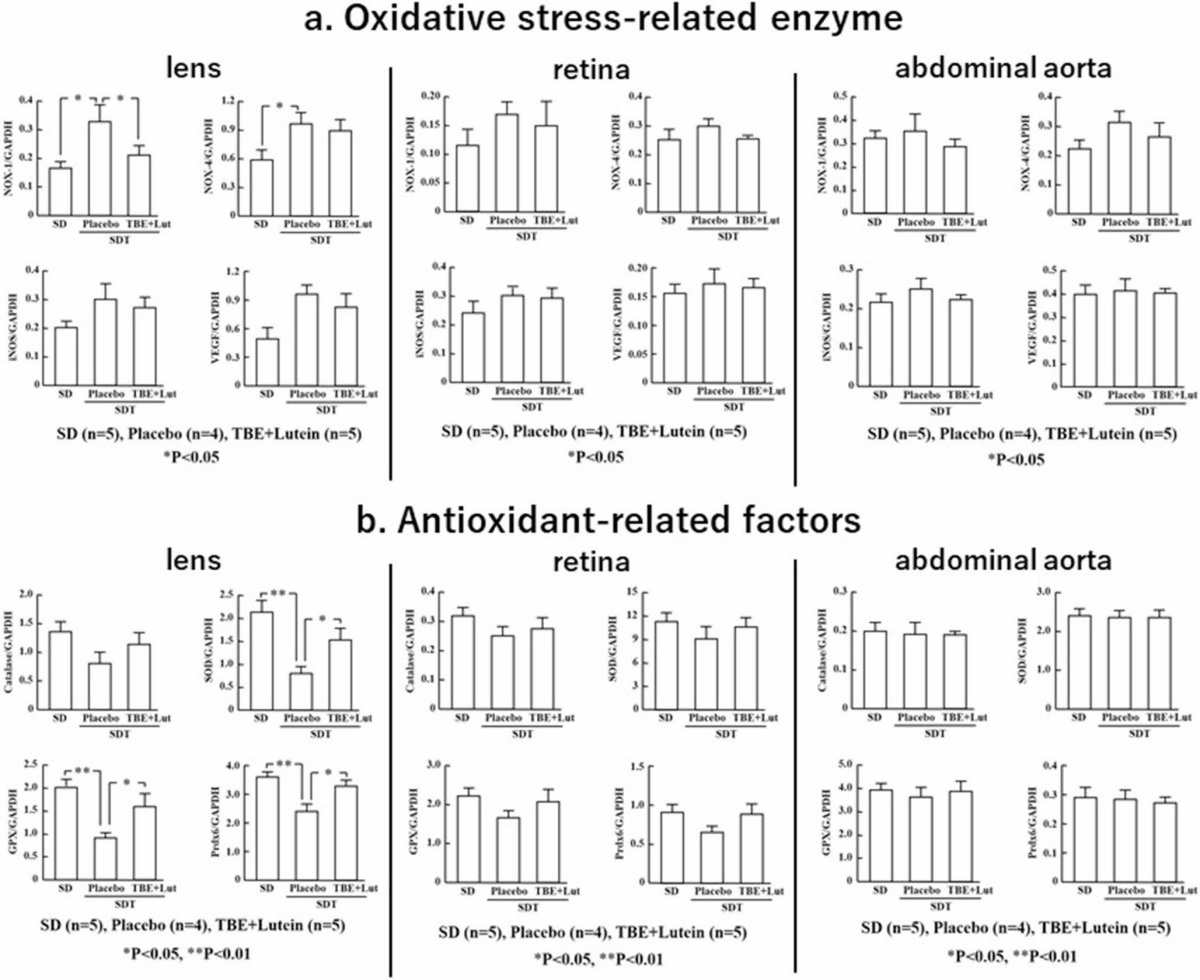
Expression of oxidative stress- and antioxidant-related genes in ocular and vascular tissues following TBE + lutein supplementation NOX-1 was significantly suppressed in the lenses of the TBE + lutein group compared to that in the placebo group (*p* < 0.05). A similar trend was observed in the retina and abdominal aorta but did not reach statistical significance. (b) SOD, GPX, and peroxiredoxin 6 were significantly increased in the lenses of the TBE + lutein group compared to those in the placebo group (*p* < 0.01). Although a similar trend was observed in the retina and abdominal aorta, it did not reach statistical significance

NOX-1 expression in the lenses was significantly higher in the placebo group than in the control group (*p* < 0.05; Fig. 3a) and was significantly suppressed in the TBE + lutein group compared to that in the placebo group (*p* < 0.05; Fig. 3a).

SOD, GPX, and peroxiredoxin 6 levels in the lenses were significantly suppressed in the placebo group compared to those in the control group (*p* < 0.05; Fig. 3b) but significantly increased in the TBE + lutein group compared to those in the placebo group (*p* < 0.01; Fig. 3b).

### Hematoxylin and eosin staining of lenses

**Fig. 4:**
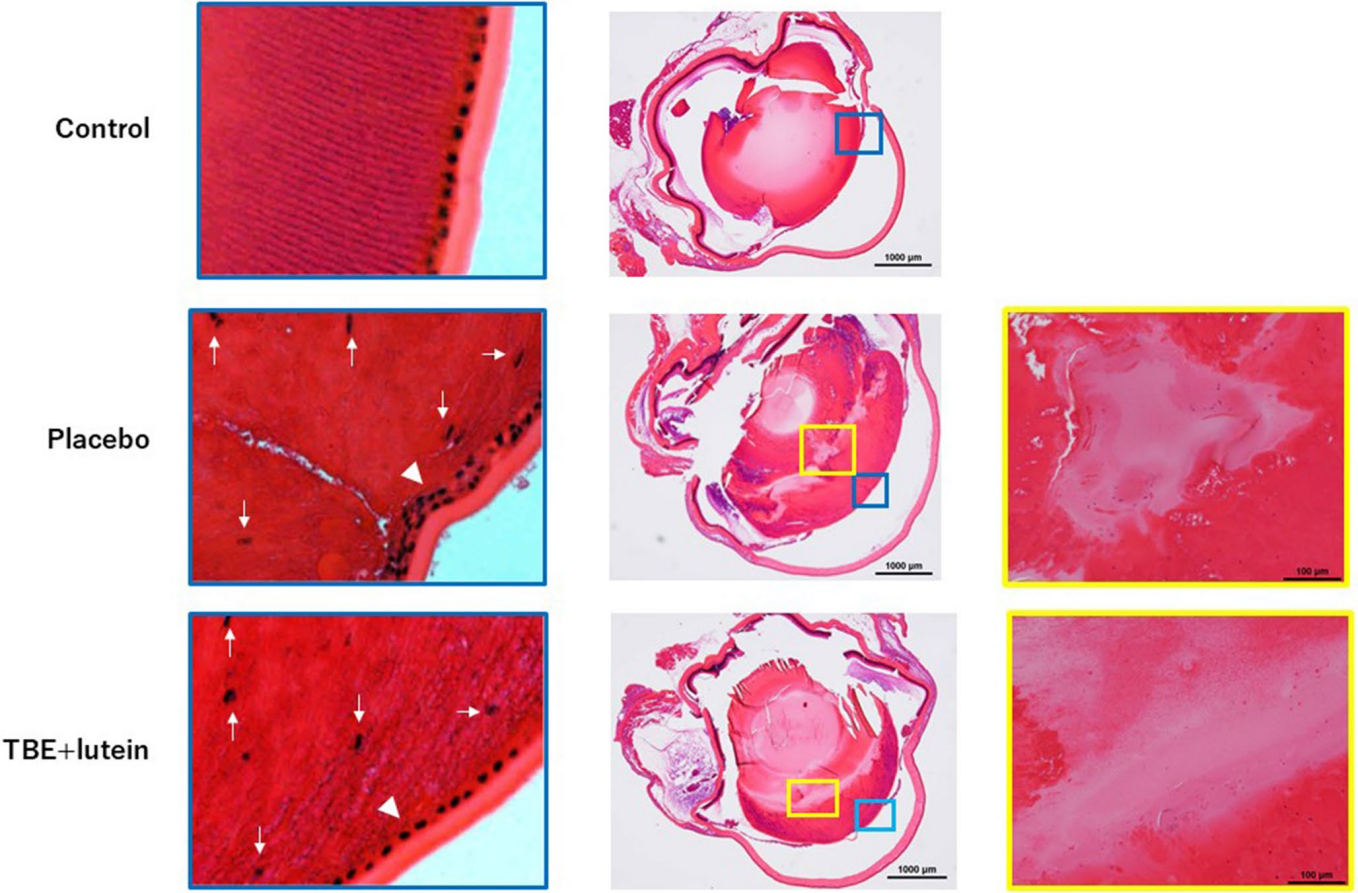
Histological analysis of lenses reveals inhibition of nuclear retention, epithelial cell proliferation, and cortical degeneration by TBE + lutein supplementation no nucleated cells were observed in the control group. In contrast, enucleation was delayed in the placebo group. This effect was inhibited in the TBE + lutein group (white arrow in blue square). The proliferation of lens epithelial cells (white arrowhead in blue square) and degeneration of cortex (yellow square) are observed in the placebo group but inhibited in the TBE + lutein group

No nucleated cells were detected in the control group (Fig. 4). In contrast, enucleation was delayed in the placebo group, where nucleated cells were still present. This effect was inhibited in the TBE + lutein group (white arrow in blue square). The proliferation of lens epithelial cells (LECs) (white arrowhead in blue square) and degeneration of the cortex (yellow square) were observed in the placebo group; however, they seemed to be decreased in the TBE + lutein group.

### Immunohistochemical staining of lenses

**Fig. 5:**
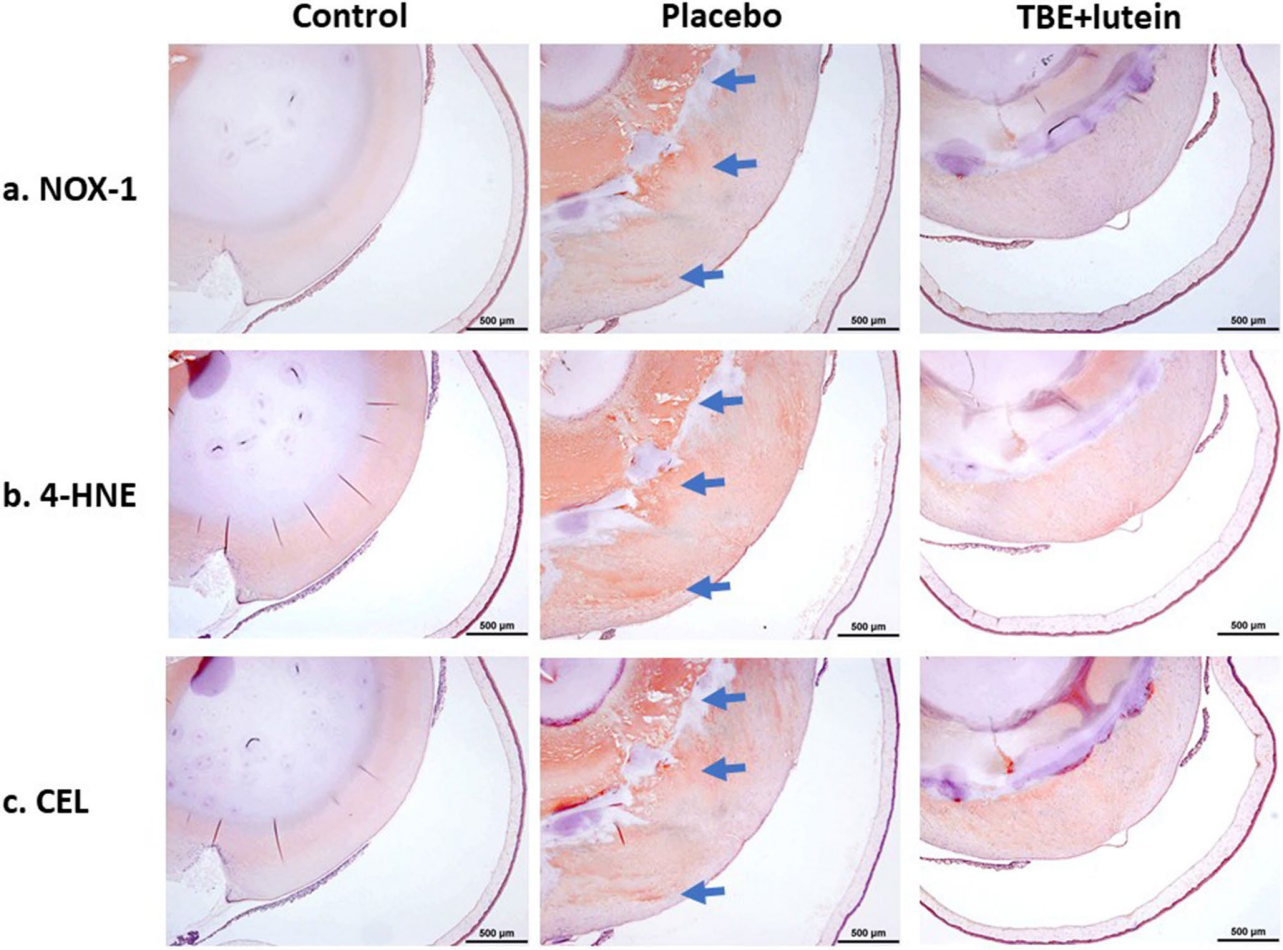
Immunohistochemical detection of oxidative stress markers (NOX-1, 4-HNE, and CEL) in lens tissues: Suppression by TBE + Lutein supplementation positive staining for NOX-1 was observed in the lens cortex and around the nucleus in the placebo group (blue arrow) and appeared to be suppressed in the TBE + lutein group (**a**). 4-HNE staining was weak in the lens cortex of the control group but became pronounced in the placebo group, particularly in the lens cortex and around the nucleus (blue arrow). This staining was reduced in the TBE + lutein group (**b**). CEL staining was absent in the control group, present in the lens cortex and around the nucleus in the placebo group (blue arrow), and suppressed in the TBE + lutein group (**c**)

Positive staining for NOX-1 was observed in the lens cortex and around the nucleus in the placebo group (blue arrow) and seemed to be suppressed in the TBE + lutein group (Fig. 5a). 4-HNE staining was weak in the lens cortex of the control group but became pronounced in the placebo group, particularly in the lens cortex and around the nucleus (blue arrow). This staining was reduced in the TBE + lutein group (Fig. 5b). CEL staining was absent in the control group, present in the lens cortex and around the nucleus in the placebo group (blue arrow), and suppressed in the TBE + lutein group (Fig. 5c).

## Discussion

Enhanced oxidative stress and AGEs are characteristic features of the hyperglycemic state in both the lenses and retinas. Excess glucose in the lenses activates the polyol pathway and promotes AGEs formation [18]. Oxidative stress causes oxidation of DNA, proteins, and lipids, leading to structural and functional abnormalities. The glycation of proteins by AGEs decreases protein solubility. High glucose levels trigger epigenetic regulation that alters gene expression in LECs. In the retina, high glucose considerably increases intracellular reactive oxygen species in both retinal microvessels and cultured microvascular cells [19, 20]. Under these hyperglycemic conditions, NOS and NADPH oxidase expression levels markedly increase in retinal microvascular endothelial cells and pericytes [19]. Suppressing both oxidative stress and AGEs is an essential step to prevent the progression of diabetic cataract.

TBE exhibits anti-glycation effects by inhibiting the glycation-induced carbonylation and cross-linking of α-crystallin [3]. Lutein absorbs light in the outer layers of the retina, suppresses lipid peroxidation, and exerts antioxidant effects by activating antioxidant enzymes, such as SOD [11, 12]. Consistent with our findings, Kinoshita et al. reported the suppression of AGEs and development of cataracts by TBE plus lutein in type 1 diabetic rats, as indicated by the considerably suppressed CML and CEL levels [9]. They reported that TBE inhibited AGEs rapidly at high concentrations, while lutein exerted inhibitory effects with long-term administration. Furthermore, the combination of TBE and lutein considerably inhibited cataract progression more than either treatment alone. Ishida et al. reported an increase in both peroxiredoxin 6 and catalase in LECs and a reduction of AGEs, leading to the prevention of cataract progression by TBE plus lutein in Shumiya cataract rats [21]. They also reported that lens opacity appeared in all rats receiving TBE plus lutein at 9 weeks of age. Hanaguri et al. reported that TBE plus lutein improved retinal blood flow, prevented glial fibrillary acidic protein activation, decreased VEGF expression in the retina, and prevented neovascularization, concluding that TBE plus lutein may prevent diabetic retinopathy [22]. Further investigation is required to clarify these effects in the retinas of SDT rats.

Slit-lamp examination of both the anterior segment of the eyes and the extracted lenses revealed inhibited cataract progression by TBE plus lutein. HE and immunohistochemical staining confirmed this effect. Although previous studies have reported cataract inhibition by TBE and lutein, to the best of our knowledge, this is the first study to simultaneously evaluate both oxidative stress-related enzymes and antioxidant-related factors using PCR. Our results showed that TBE plus lutein had a stronger effect on antioxidant-related factors than on oxidative stress-related enzymes in the lenses, suggesting a greater impact on enhancing the antioxidant defense system. This provides new insights into the potential mechanism by which TBE plus lutein inhibits cataract progression, which has not been previously reported.

The present study has some limitations. Although trends were observed—such as stronger effects of TBE plus lutein on antioxidant-related factors—many of these differences did not reach statistical significance, limiting the strength of the conclusions. Additionally, only 15 rats were included in the study group, which may have limited the ability to detect statistically significant differences. Notably, most evaluations, particularly those using PCR, were performed at a single time point. Furthermore, we examined the combination of TBE and lutein but did not include groups receiving TBE or lutein alone. This makes it difficult to determine the individual contribution of each compound. Longitudinal data would provide a better understanding of the progression and timing of TBE plus lutein’s effects. In this study, the compound was not administered via forced feeding. Moreover, the present study investigated only mRNA expression related to antioxidant activity and oxidative stress-associated enzymes. Changes in NOX-1, 4-HNE, and CEL expression were assessed only using immunohistochemical staining, without protein-level such as via western blotting. This is due to the fact that the protein yield from rat lenses was insufficient to permit analyses beyond genetic evaluation. Further studies are therefore required to quantify protein levels and assess enzymatic activity.

Additionally, TBE plus lutein suppressed the enhanced oxidative stress and AGEs induced by hyperglycemia in the lenses of diabetic rats and had a stronger effect on antioxidant-related factors than on oxidative stress-related enzymes in diabetic cataracts. Finally, lens opacity caused by oxidative stress developed earlier than changes in the retina and aorta. Furthermore, TBE plus lutein may inhibit the enhanced oxidative stress in the retina and systemic macrovessels.

In conclusion, TBE plus lutein may help prevent diabetic cataract by enhancing antioxidant defenses and suppressing AGEs. These findings support its potential as a complementary therapy; however, further studies are warranted to confirm its efficacy and clarify the underlying mechanisms.

## Data Availability

No datasets were generated or analysed during the current study.
